# Bartonellosis in World Health Organization Eastern Mediterranean Region, a systematic review and meta-analysis

**DOI:** 10.1093/eurpub/ckae123

**Published:** 2025-01-13

**Authors:** Zahra Tahmasebi Ashtiani, Fahimeh Bagheri Amiri, Mozhgan Ahmadinezhad, Ehsan Mostafavi, Saber Esmaeili

**Affiliations:** Department of Epidemiology and Biostatistics, Research Centre for Emerging and Reemerging Infectious Diseases, Pasteur Institute of Iran, Tehran, Iran; National Reference Laboratory of Plague, Tularemia and Q Fever, Research Centre for Emerging and Reemerging Infectious Diseases, Pasteur Institute of Iran, Hamadan, Iran; Department of Epidemiology and Biostatistics, Research Centre for Emerging and Reemerging Infectious Diseases, Pasteur Institute of Iran, Tehran, Iran; Department of Epidemiology and Biostatistics, Research Centre for Emerging and Reemerging Infectious Diseases, Pasteur Institute of Iran, Tehran, Iran; Department of Epidemiology and Biostatistics, Research Centre for Emerging and Reemerging Infectious Diseases, Pasteur Institute of Iran, Tehran, Iran; National Reference Laboratory of Plague, Tularemia and Q Fever, Research Centre for Emerging and Reemerging Infectious Diseases, Pasteur Institute of Iran, Hamadan, Iran; Department of Epidemiology and Biostatistics, Research Centre for Emerging and Reemerging Infectious Diseases, Pasteur Institute of Iran, Tehran, Iran; National Reference Laboratory of Plague, Tularemia and Q Fever, Research Centre for Emerging and Reemerging Infectious Diseases, Pasteur Institute of Iran, Hamadan, Iran

## Abstract

*Bartonella* is a vector-borne zoonotic pathogen, which could also be transmitted directly and cause a variety of clinical illnesses. This study aimed to investigate the prevalence of *Bartonella* in countries in the WHO Eastern Mediterranean Region (WHO-EMR) region. We searched using the keywords *Bartonella* and the name of each country in the WHO-EMR in databases such as PubMed, ISI (Web of Science), Scopus, and Google Scholar, with a publication date range of 1990–2022 and limited to English articles. We evaluated the quality of the studies using the STROBE 6-item checklist and used the random effects model to integrate the findings of the included studies. A total of 45 papers out of 240 were included in the analysis. The results showed the prevalence of Bartonella infection among endocarditis patients was 3.8% (95% CI: 0.2–7.4) and the seroprevalence of *Bartonella* among other people was 27.5% (95% CI: 13.5–41.5). The overall prevalence of *Bartonella* spp. among animals, as determined by molecular, serological, and culture methods, was 11.9% (95% CI: 5.7–18.2), 38.9% (95% CI: 27.5–50.2), and 1.7% (95% CI: 0.5–2.9), respectively. Furthermore, the prevalence of *Bartonella* spp. in ectoparasites was 3.9% (95% CI: 3.5–5.2), with fleas (6.2%) showing a higher prevalence compared to lice (4.9%) and ticks (1.0%). The detection of *Bartonella* in all animal and ectoparasites species and human populations in the WHO-EMR with prevalence ranging from 0.3% to 23% is concerning, emphasizes the importance of conducting more comprehensive studies to gain a deeper understanding of the spread of *Bartonella* in these areas.

Key points

- Bartonellosis is a neglected tropical disease in the WHO-EMR and there is a gap in data about the status of *Bartonella* infection in animal hosts, vectors, and humans.
- The prevalence of *Bartonella* in animals was higher than in humans and ectoparasites.
- Rodents had the most prevalence among all animals.
- The prevalence of *Bartonella* spp. in fleas was higher than in lice and ticks.

## Introduction

Bartonellosis is an emerging infectious disease caused by *Bartonella* spp. *Bartonella* is a gram-negative bacterium that is known as a zoonotic pathogen. While fleas are the primary vector for different *Bartonella* species in nature, transmission of the bacterium to mammalian hosts can also occur through other arthropods such as lice, sand flies, mites, and ticks [1]. This bacterium is highly adaptable to its reservoirs and hosts, and it can infect a wide range of domestic and wild animals, including cats, dogs, and rodents, as well as humans. *Bartonella* can persist in hosts for extended periods, creating numerous natural reservoirs that can potentially lead to human infections [2]. Until 2021, at least 20 *Bartonella* species of 53 species have been reported to infect humans [3–4].

The severity of bartonellosis in humans is closely linked to the immune status of the individual, and immunocompromised patients, such as HIV-positive patients and organ transplant recipients, are at a greater risk of infection and severe complications compared to healthy individuals. Previous studies conducted in Spain and America have reported a prevalence of *Bartonella* antibodies ranging from 5.88% to 24.7% among high-risk populations such as HIV-positive individuals and injection drug users [5].

The low-income status of many countries in the WHO-EMR makes *Bartonella* infection a potential threat to their health systems. Furthermore, studies have shown that this disease has become a significant health challenge for humans and animals in the WHO-EMR [6–8]. As we know, there is no general estimate of the prevalence of *Bartonella* infection in the population of the WHO-EMR. This study aimed to estimate the overall prevalence of *Bartonella* among humans, animals, and ectoparasites in the WHO-EMR using a systematic review and meta-analysis study.

## Methods

### Search strategy

We performed a comprehensive search in four electronic databases, namely PubMed, SCOPUS, Web of Science, and Google Scholar, covering the period from 1990 to 2022. We defined search terms as “*Bartonella*” or “*Bartonella* infection” and “Epidemiology” or “Prevalence” or “Frequency” and each one of the following countries: Pakistan, Afghanistan, Bahrain, Djibouti, Egypt, Iran, Iraq, Jordan, Kuwait, Lebanon, Libya, Morocco, Oman, Qatar, Somalia, Saudi Arabia, Syria, Sudan, Tunisia, United Arab Emirates, Yemen, and Palestine.

#### Study selection

Initially, two independent reviewers (Z.T.A. and M.A.) conducted a review of the titles and abstracts of articles to identify the relevant studies. Subsequently, they examined the full text of eligible articles. We resolved conflicts arising upon the selection of studies by a third reviewer (S.E.).

#### Eligibility criteria

In this study, we defined inclusion criteria as: articles that reported on the prevalence of various *Bartonella* species in humans, animals, and ectoparasites in the countries of interest, and published articles in English between 1990 and 2022. Although we defined exclusion criteria as: published studies as review, letters to editor, case reports, case series, as well as books, or studies that lacked relevant information, including sample size and positive cases.

#### Data collection and data items

We extracted relevant data such as place, date of study, sample size, number of positive samples, method of test, kind of sample, reported *Bartonella* species, and study population (animals, humans, or ectoparasites) from the articles and recorded in Microsoft Excel. Two independent researchers (Z.T.A. and M.A.) conducted the data extraction, and a third researcher (F.B.A.) reviewed the information to ensure its accuracy.

#### Quality evaluation

We controlled the bias primarily by the use of inclusion and exclusion criteria. We evaluated the quality of eligible articles using the modified STROBE checklist. Two independent reviewers assessed each article’s quality based on the checklist (M.A. and Z.T.A.) and in cases of disagreement, a third reviewer (F.B.A. and S.E.) re-evaluated the article. The checklist items assessed included eligibility criteria, clear outcome definition, location description, disease species definition, sample size, and number of outcomes reported. For each item, one point was assigned, resulting in a maximum score of 6 points for good-quality studies. We categorized the selected studies based on their scores, with a score of 5 or 6 considered high quality, a score of 3 or 4 considered medium quality, and a score of less than 3 considered low quality.

#### Analytic approach

We used Stata software (version 17) for conducting meta-analysis, which involved separate analyses for assessing the prevalence of *Bartonella* spp. in animals, humans, and ectoparasites. We carried out subgroup analyses based on detection method, animal and ectoparasites species, and region if there were at least two studies in each subgroup. We reported the pooled prevalence and 95% confidence intervals using Metaprop command [9], *Q* statistic with the Chi-square and *P* values, and the *I*^2^ statistic as a percentage. The DerSimonian-Laird method was used as the random effects [10].

## Results

### Search result

**Figure 1.**
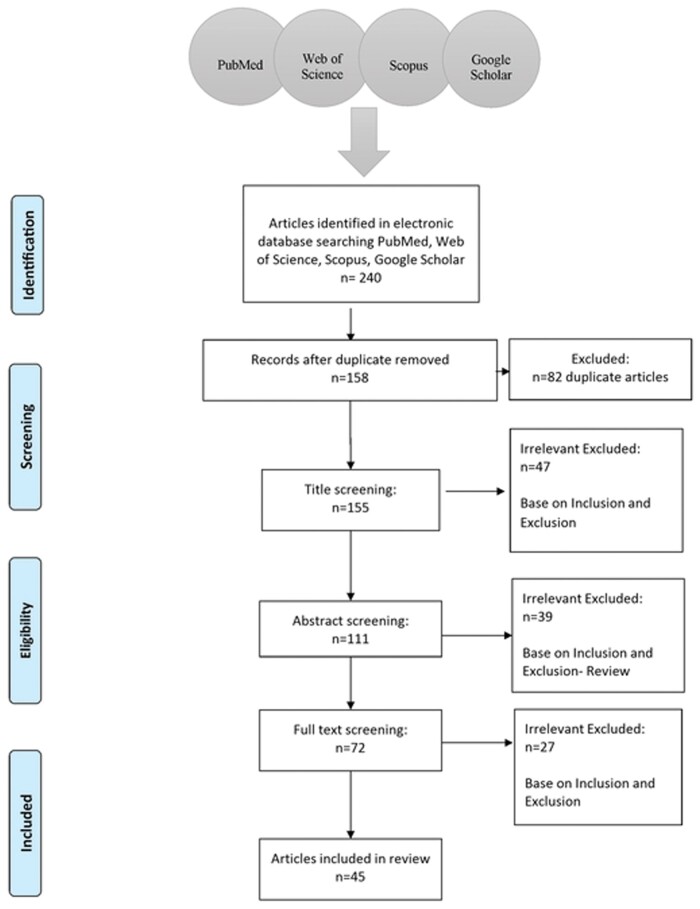
Flowchart for the selection of the studies included in the systematic review and meta-analysis of Bartonella infections in WHO-EMR countries, 1990–2022.

The result of this systematic review and meta-analysis was reported according to the 2020 Preferred Reporting Items for Systematic Reviews and Meta-Analyses (PRISMA) checklist [11]. The initial search identified 240 studies in the WHO-EMR and we finally included 45 articles in this study (Fig. 1).

Iran, Egypt, Tunisia, Morocco, Jordan, Saudi Arabia, Palestine, Iraq, Pakistan, Lebanon, Bahrain, and Afghanistan included studies on humans, animals, and ectoparasites (Supplementary Table S1).

### Quality assessment

According to the quality assessment, 35 studies (77.7%) had an overall rating of 5–6 and good quality, while 10 studies (22.2%) had an overall rating of 3–4 and were of moderate quality according to the classification. There were no studies with poor quality among the included studies (Supplementary Table S2).

### *Bartonella* spp. in human

In total, we found 10 studies on different human populations. To better describe the results, we categorized human studies based on the study population; so, we defined two main groups: (i) endocarditis patients, and (ii) other groups as fairly asymptomatic people. We did separate meta-analysis for endocarditis patients as a symptomatic group (*n* = 5), and on other groups as fairly asymptomatic people (*n* = 4).

#### Prevalence of *Bartonella* spp. in endocarditis patients

There was a total of five studies that carried out endocarditis patients (*n* = 705 samples). The total prevalence of *Bartonella* spp. in endocarditis patients was 3.8% (95% CI: 0.2–7.4). There were five reports using molecular methods (three on blood and two on valve) and five reports using serological methods.

##### Valve samples

The prevalence of *Bartonella* spp. in valve samples based on two studies (*n* = 90 valve) was 3.3% (95% CI: 0.0–7.0).

##### Blood samples

**Figure 2.**
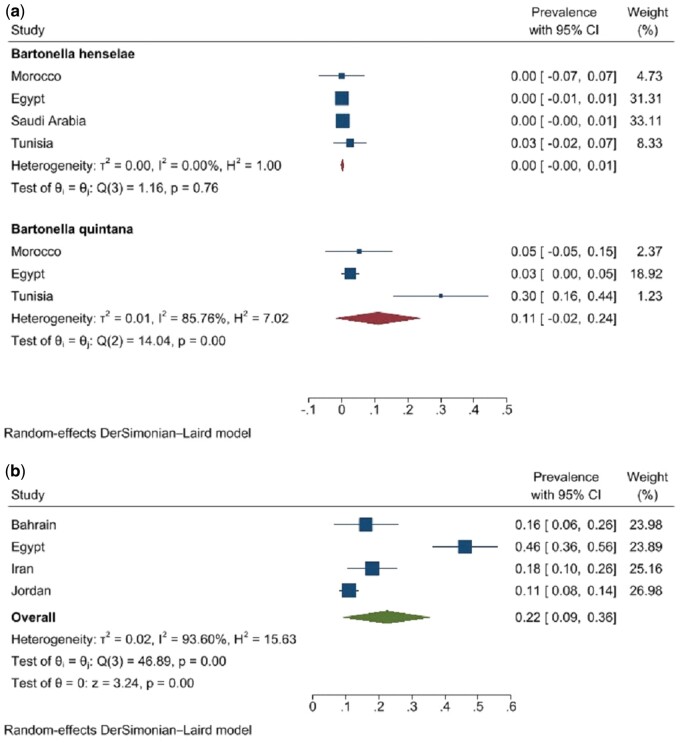
Prevalence of detected *Bartonella* species in endocarditis patients (a) and other groups (b), in WHO-EMR countries, 1990–2022.

All five studies on endocarditis patients reported prevalence using serological methods, and three reported it based on the PCR method. The seroprevalence and prevalence using a molecular assay in endocarditis patients were 7.2% (95% CI: 0.0–18.1) and 3.8% (0.2%–7.4%, *I*^2^: 84.82%), respectively. The detected species using the serological method included *B. quintana* (11.0%, 95% CI: 0.0–24.2) and *B. henselae* (0.2%, 95% CI: 0.0–0.7) (Fig. 2a).

#### Prevalence of *Bartonella* spp. on other groups (fairly asymptomatic people)

There were only four seroprevalence studies on cat owners, HIV-positive patients, and hospitalized children (*n* = 738). Total prevalence was 27.5% (95% CI: 13.5–41.5, *I*^2^: 92.03%). Subgroup analysis on cat owners (*n* = 352 people from three studies) showed an infection rate of 28.4% (95% CI: 13.5–56.3, *I*^2^: 90.7%). The subgroup analysis on HIV-positive and hospitalized children was not applicable, because there is only one study in each subgroup.

The prevalence of *B. henselae* in all seroprevalence studies was 22.0% (95% CI: 9.0–36.0), and only one study detected *B. quintana* (Fig. 2b).

### *Bartonella* spp. in animal

#### Prevalence of *Bartonella* based on molecular assay

We assessed a total of 3728 samples of different species of animals from 21 articles for *Bartonella* spp. prevalence using PCR assay. The pooled molecular prevalence of *Bartonella* spp. among all species of animals in this region was 11.9% (95% CI: 5.7–18.2, *I*^2^: 99.35 and *P* < .001). Among the countries, Tunisia and Saudi Arabia had the lowest (6.4%, 95% CI: 0.0–12.8) and highest (36.2%, 95% CI: 0.00–98.6) prevalence among animals, respectively. African countries (7.6%) had a lower prevalence in comparison to Asian countries (14.1%). Based on subgroup analysis, the rodents (23.0%), cats (12.2%), and dogs (8.1%) had the highest prevalence of *Bartonella* spp. The prevalence of *Bartonella* in ruminants (5.9%) was higher than in camels (2.4%) (Supplementary Fig. S1). There was only one study on wild animals and one study on equines. Only 11 studies reported the *Bartonella* species.

The molecular prevalence of *B. henselae*, *B. vinsonii*, and *B. merieuxii* in animals was 3.7% (95% CI: 1.6–5.8), 3.3% (95% CI: 0.2–6.4), and 17.6% (95% CI: 0.0–35.9), respectively (Table 1).

**Table 1.** Subgroup analysis of *Bartonella* spp. prevalence in mammals and ectoparasites of the WHO-EMR countries, 1990–2022

	Total studies	Total sample	Pooled prevalence (95% CI)	*I* ^2^
Animal (molecular methods)				
Animal group				
Ruminant	2	214	5.9 (2.7–9.2)	0.00
Camel	5	837	2.4 (0.0–5.0)	88.43
Cat	6	505	12.2 (5.5–18.9)	83.35
Dog	8	822	8.1 (4.5–11.7)	93.89
Rodent	4	1424	23.0 (7.2–38.9)	98.82
Country				
Iran	9	980	8.7 (3.2–14.2)	95.20
Egypt	3	1395	7.2 (5.8–8.5)	00.05
Tunisia	3	661	6.4 (0.0–12.8)	94.59
Saudi Arabia	2	252	36.2 (0.0–98.6)	99.52
Others^a^	4	640	13.3 (2.9–23.8)	95.45
Kind of sample				
Blood	29	3520	6.5 (4.7–8.3)	90.66
Nail	3	310	0.5 (0.00–2.1)	62.21
Saliva	3	310	2.7 (0.00–6.6)	83.74
Tissue	2	238	35.0 (0.00–99.7)	99.60
Continent				
African countries	7	2344	7.6 (4.2–11.0)	89.93
Asian countries	14	1584	14.1 (4.8–23.4)	99.57
Quality				
Moderate	5	719	19.6 (10.7–28.4)	98.97
High	19	3629	11.6 (7.9–15.3)	96.83
Species				
*B. henselae*	8	947	3.7 (1.6–5.8)	64.99
*B. vinsonii*	2	162	3.3 (0. 2–6.4)	77.81
*B. merieuxii*	3	259	17.6 (0.0–35.9)	94.23
Animal (serological methods)				
Animal group				
Cat	4	638	35.7 (20.-51.3)	94.67
Dog	4	495	44.5 (22.6–66.5)	96.31
Country				
Iran	2	166	48.5 (0.0–98.7)	98.22
Iraq	2	400	32.8 (22.2–43.4)	81.25
Others^b^	4	627	37.4 (20.6–66.2)	95.43
Continent				
African countries	3	474	38.8 (15.4–62.2)	96.92
Asian countries	5	719	38.9 (24.9–52.9)	93.32
Quality				
Moderate	2	340	38.2 (33.1–43.4)	0.00
High	6	853	39.2 (23.6–54.7)	96.23
Species				
*B. henselae*	5	657	27.9 (14.0–41.7)	95.48
*B. vinsonii*	2	215	20.3 (15.6–24.9)	98.57
*B. clarridgeiae*	2	422	24.5 (6.9–42.1)	96.22
Ectoparasites				
Continent				
African countries	6	2829	4.0 (1.4–6.6)	96.92
Asian countries	7	3433	4.3 (2.1–6.5)	95.18
Class of ectoparasites				
Arachnida	4	1262	1.0 (0.0–2.3)	87.55
Insecta	10	5000	6.1 (3.7–8.5)	96.45
Order				
Flea	8	4817	6.2 (3.6–8.9)	97.09
Tick	4	1262	1.0 (0.0–2.3)	87.55
Lice	2	183	4.9 (0.0–13.9)	80.88
Family				
Ixodoidea	4	1262	1.0 (0.0–2.3)	87.55
Pulicidea	5	3696	7.4 (3.0–11.9)	98.06
Genus				
*Ctenocephalides*	5	2478	7.9 (2.8–13.0)	97.76
*Hyaloma*	3	603	0.3 (0.0–1.2)	58.82
*Pulex*	3	1183	0.5 (0.0–2.2)	80.94
*Rhipicephalus*	2	604	2.0 (0.0–6.1)	92.26
*Xenopsylla*	2	906	26.5 (0.0–80.0)	88.39
Species of *Bartonella*				
*B. henselae*	5	1366	5.3 (2.2–8.5)	87.76
*B. clarridgeiae*	5	994	3.3 (0.1–6.5)	90.84
*B. rattimassiliensis*	2	163	8.1 (3.5–12.7)	17.34
*B. rochalimae*	3	461	2.7 (1.2–4.1)	0.00
*B. koehlerae*	3	203	2.0 (0.1–4.0)	0.00

The results of animals are shown in parts based on molecular methods and serological methods.

a Including Iraq, Palestine, Morocco, Jordan, and Pakistan.

b Including Jordan, Egypt, Morocco, and Tunisia.

#### Serological prevalence in animals

Based on the result of 1193 samples from eight studies in the WHO-EMR countries, the total seroprevalence of *Bartonella* among animals was 38.9% (95% CI: 27.5–50.2, *I*^2^: 94.76, *P* < .001). All seroprevalence studies focused on cats, dogs, and wild animals (only one study). The seroprevalence in cats and dogs was 35.7% and 44.5%, respectively. The seroprevalence of *Bartonella* spp. in African countries (38.8%) was almost similar to Asian countries (38.9%). Furthermore, the seroprevalence of *B. henselae*, *B. vinsonii*, and *B. clarridgeiae* was 27.9% (95% CI: 14.0–41.7), 20.3% (15.6–24.9), and 24.5% (95% CI: 6.9–42.1), respectively (Table 1).

#### Culture

We found only four studies using the culture method. The total prevalence of *Bartonella* bacterium was 1.7% (95% CI: 0. 5–2.9). The subgroup analysis showed a high prevalence in rodents (15.4%, 95% CI: 10.4–20.5) and a lower prevalence in cats (0.8%, 95% CI: 0.0–2.1).

### *Bartonella* in ectoparasites

The meta-analysis of 13 studies on 6262 ectoparasites in WHO-EMR countries revealed that the estimated prevalence of *Bartonella* spp. is 3.9% (95% CI: 3.5–5.2). The prevalence of *Bartonella* in ectoparasites in Africa (4.0%) was the same as in ectoparasites of Asian countries (4.3%) (Table 1 and Supplementary Fig. S2). The prevalence of *Bartonella* spp. in fleas (6.2%, 95% CI: 3.6–8.9) was higher than in lice (4.9%, 95% CI: 0.0–13.9) and ticks (1.0%, 95% CI: 0.0–2.3) (Supplementary Fig. S2), the genus of *Xenopsylla* (26.5%, 95% CI: 0.0–80.0) and *Ctenocephalides* (7.9%, 95% CI: 2.8–13.0) showed highest prevalence (Supplementary Fig. S3).

## Discussion

The present study aimed to investigate the prevalence of *Bartonella* considering One Health view in WHO-EMR, which includes 15 Asian and seven African countries. In this study, 45 articles from 12 countries, showed evidence of current infection or history of exposure in humans, animals, and ectoparasites. In the 10 other countries including Djibouti, Kuwait, Libya, Oman, Qatar, Somalia, Sudan, Syrian Arab Republic, United Arab Emirates, and Yemen, we didn’t find any eligible article. One Health is an approach that considers people's health close to the health of animals and the environment [12].

The prevalence of *Bartonella* using a molecular assay in humans, animals, and ectoparasites was 0.4%, 11.9%, and 3.9%, respectively. Total serologic prevalence in humans and animals was 13.7% and 38.9%, respectively. Higher prevalence in animals rather than humans and ectoparasites may emphasize the role of animals as reservoirs.

Totally, the prevalence of *Bartonella* spp. in people with endocarditis based on all types of samples was more than 3%. Some zoonosis bacteria like *Bartonella* are responsible for endocarditis and at least eight species of *Bartonella* have been recognized as causing infective endocarditis in humans including: *B. quintana* and *B. henselae* (in 95% of cases), and other species in 5% included *B. elizabethae*, *B. vinsonii*, *B. koehlerae*, *B. clarridgeiae*, *B. washoensis*, and *B. alsatica* [13–17]. In this study, we only found reports of *B. quintana* (11%) and *B. henselae* (0.2%) in serum of endocarditic patients.

The seroprevalence in cat owners (28.4%) was higher than in endocarditis patients (7.2%). The domestic cat is the major reservoir for some *Bartonella* species, especially *B. henselae* and cat fleas are the primary vector of feline *Bartonella* [12].

Among animals, the highest prevalence of *Bartonella* was in rodents (23.0%), cats (12.2%), and dogs (8.1%). The prevalence of *Bartonella* in ruminants (5.9%) was higher in camels (2.4%). As the literature shows, *Bartonella* spp. are highly adapted to one or few mammalian reservoir hosts [2]. Based on our results, rodents seem to be potential reservoirs for *Bartonella* species in WHO-EMR countries. As previous study showed, the rodents are associated with at least 20 of all *Bartonella* species [18].

The prevalence of *Bartonella* spp. in collected ectoparasites was 3.9%. The prevalence of *Bartonella* spp. in fleas was higher than other ectoparasites. The genus of *Xenopsylla* and *Ctenocephalides* had the highest prevalence. Blood-sucking arthropods, such as sandflies (*Lutzomyia verrucarum*), human lice (*Pediculus humanus*), cat fleas (*Ctenocephalides felis*), some rodent fleas (*Ctenophthalmus nobilis*), and ticks have been reported as potential vectors transmitting *Bartonella* species [19]. Fleas, especially the *Xenopsylla cheopis*, known as rat fleas, are also considered to be the main vectors of *Bartonella* infection [20].

Unfortunately, only a few studies reported the *Bartonella* species. The most commonly reported species-based molecular and serological tests in animals were *B. merieuxii* (17.6%) and *B. henselae* (27.9%). However, the main pathogenic species of *Bartonella* for humans included *B. henselae*, *B. quintana*, and *B. bacilliformis*, but some other species including *B. melophagi*, *B. rattimassiliensis*, *B. rochalimae*, *B. alsatica*, *B. ancashensis*, *B. clarridgeiae*, *B. grahamii*, *B. koehlerae*, *B. kosoyi*, *B. mayotimonensis*, *B. schoenbuchensis*, *B. tamiae*, *B. tribocorum*, *B. doshiae*, *B. elizabethae*, *B. vinsonii*, and *B. washoensis* are described as a cause of disease with mild to severe symptoms, especially in immunocompromised patients [21]. *Bartonella henselae* and *B. quintana* are frequent causes of illness in immunocompromised people that often go undiagnosed due to, it being a neglected disease in many countries. Furthermore, sometimes it remains undiagnosed because of the technical challenges for *Bartonella* species.

In this study, all animals, ectoparasites, and humans showed evidence of infection with *Bartonella* spp., which the rodents, cats, dog, genus of *Xenopsylla*, and *Ctenocephalides* had highest prevalence. This finding shows the impact of considering One Health approach for controlling pathogens such as *Bartonella* spp. Climatic conditions are related to the prevalence of *Bartonella* infections in animals (based on breeding season, which causes more reservoirs), and the penetration of ectoparasites infestations is greater in warm and humid conditions [22]. Considering that *Bartonella* species have the ability to infect and survive within erythrocytes, resulting in prolonged intra-erythrocytic and potentially intra-endothelial infections, which may be associated with a recurrent pattern of bacteremia. This can lead to subclinical bloodstream infections in humans. Prolonged bacteremia increases the likelihood of transmission between hosts through arthropod vectors and other modes of transmission [23]. Based on researches *henselae* can survive in stored blood for 35 days [24], as well as after inoculation of the *B. henselae* into blood, it adhered to human erythrocytes (after 10 h) and intraerythrocytic infection (after 72 h) [25]. These results suggested a potentially important role for *Bartonella* sp. in transfusion medicine [23].

Considering that *Bartonella* does not have severe symptoms in healthy people and based can only survive in erythrocytes, as a result, the importance of infection in this group of people increases when they can somehow transmit the infection to people with an incomplete immune system. One of the important roots of transmission is donated bloods, because almost all people in need of blood transfusion, due to underlying problems, such as childbirth, accidents, congenital diseases, etc., have a weak immune system, so receiving infected blood may make some severe symptom. So having a surveillance program for assessing *Bartonella* infections in blood donors, who are apparently healthy, could be helpful for controlling the transmission of infection to people who needs blood and are at high risk of progress sever symptoms after infection.

## Conclusion

Bartonellosis is an emerging infectious disease. Our systematic review found only 45 studies on Bartonellosis across 12 WHO EMR countries during 1990–2022. Besides no studies from 10 countries, we also found few studies using One Health approach, considering at least vector and host. Countries and international agencies should invest more in research and surveillance to understand the epidemiology of Bartonellosis and to inform the health systems and physicians.

## Supplementary Material

ckae123_Supplementary_Data

## Data Availability

The original contributions presented in the study are included in the article/supplementary material, and further inquiries can be directed to the corresponding authors.
