# Supplementary material for: Bartonellosis in World Health Organization Eastern Mediterranean Region, a systematic review and meta-analysis
Source: Eur J Public Health. 2025 Jan 13;35(Suppl 1):i48–54. doi: 10.1093/eurpub/ckae123 (PMC11725950; doi:10.1093/eurpub/ckae123)
Supplement: ckae123_Supplementary_Data [file ckae123_supplementary_data.pdf]

# Supplementary data

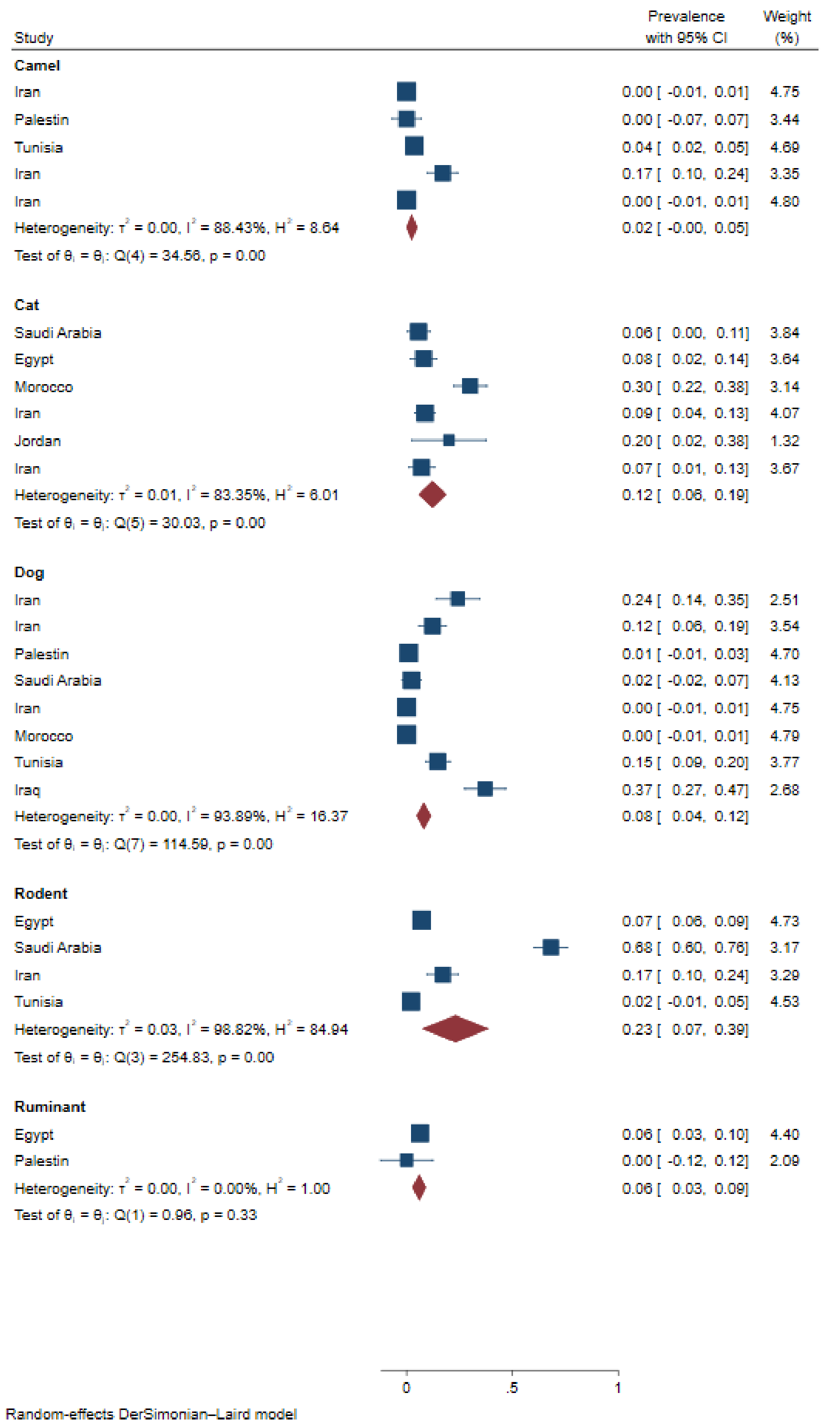


**S1 Figure. The prevalence of Bartonella spp. in animal samples of EMRO-WHO countries based on animal species, a systematic review and meta-analysis of 25 studies in the nine countries (1990-2022).**

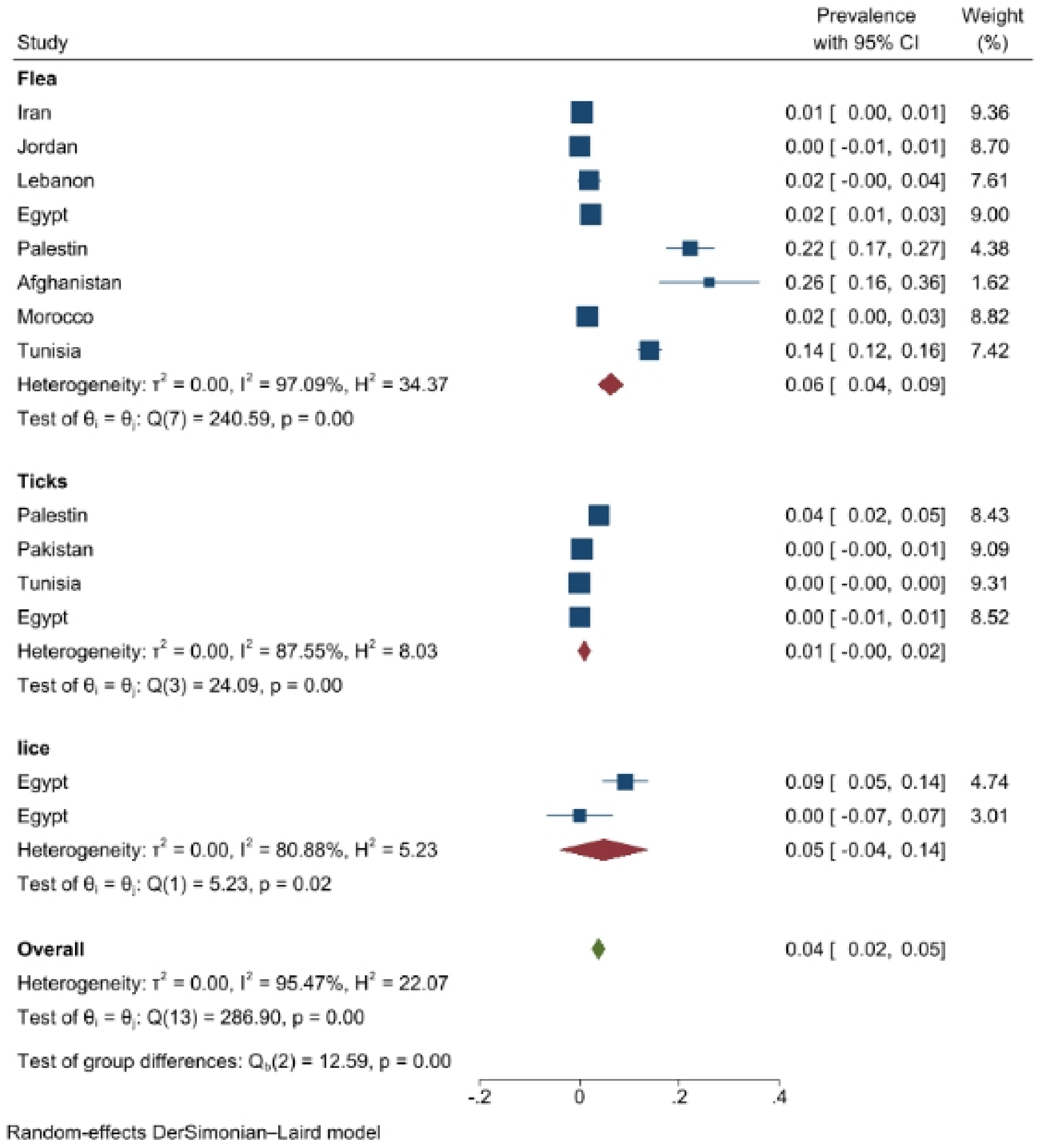


**S2 Figure. The prevalence of Bartonella spp. in ectoparasites of EMRO-WHO countries based on different orders of ectoparasites, a systematic review and meta-analysis of 14 studies in the nine countries (1990-2022).**

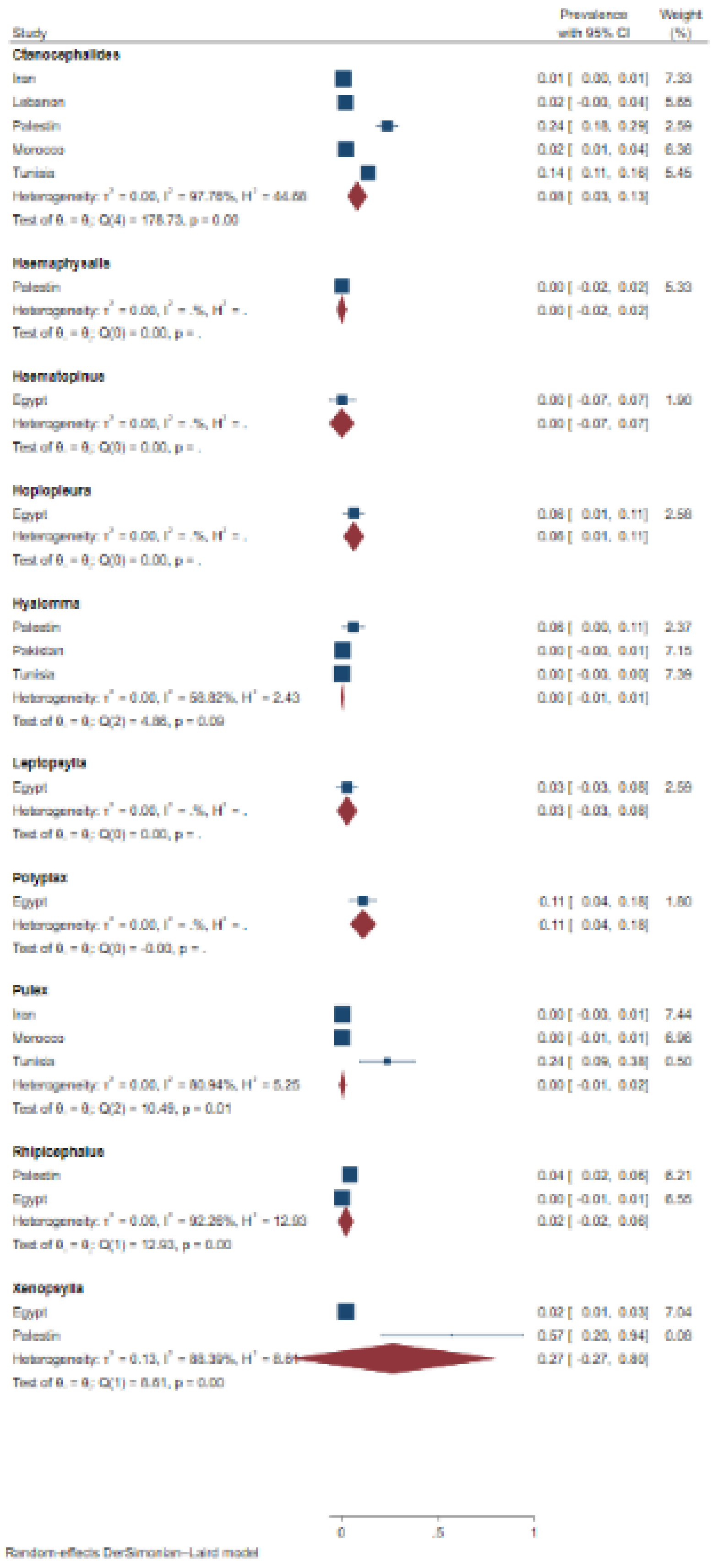

Study
Prevalence with 95% CI
Weight (%)
Ctenocephalides
Iran 0.01 [ 0.00, 0.01] 7.33
Lebanon 0.02 [ -0.00, 0.04] 5.65
Palestin 0.24 [ 0.18, 0.29] 2.59
Morocco 0.02 [ 0.01, 0.04] 6.36
Tunisia 0.14 [ 0.11, 0.16] 5.45
Heterogeneity: $\tau^2$ = 0.00, $I^2$ = 97.76%, $H^2$ = 44.68 0.08 [ 0.03, 0.13]
Test of $\theta_i = \theta_j$: Q(4) = 178.73, p = 0.00
Haemaphysalis
Palestin 0.00 [ -0.02, 0.02] 5.33
Heterogeneity: $\tau^2$ = 0.00, $I^2$ = .%, $H^2$ = . 0.00 [ -0.02, 0.02]
Test of $\theta_i = \theta_j$: Q(0) = 0.00, p = .
Haematopinus
Egypt 0.00 [ -0.07, 0.07] 1.90
Heterogeneity: $\tau^2$ = 0.00, $I^2$ = .%, $H^2$ = . 0.00 [ -0.07, 0.07]
Test of $\theta_i = \theta_j$: Q(0) = 0.00, p = .
Hoplopleura
Egypt 0.06 [ 0.01, 0.11] 2.58
Heterogeneity: $\tau^2$ = 0.00, $I^2$ = .%, $H^2$ = . 0.06 [ 0.01, 0.11]
Test of $\theta_i = \theta_j$: Q(0) = 0.00, p = .
Hyalomma
Palestin 0.06 [ 0.00, 0.11] 2.37
Pakistan 0.00 [ -0.00, 0.01] 7.15
Tunisia 0.00 [ -0.00, 0.00] 7.39
Heterogeneity: $\tau^2$ = 0.00, $I^2$ = 58.82%, $H^2$ = 2.43 0.00 [ -0.01, 0.01]
Test of $\theta_i = \theta_j$: Q(2) = 4.86, p = 0.09
Leptopsylla
Egypt 0.03 [ -0.03, 0.08] 2.59
Heterogeneity: $\tau^2$ = 0.00, $I^2$ = .%, $H^2$ = . 0.03 [ -0.03, 0.08]
Test of $\theta_i = \theta_j$: Q(0) = 0.00, p = .
Polyplax
Egypt 0.11 [ 0.04, 0.18] 1.80
Heterogeneity: $\tau^2$ = 0.00, $I^2$ = .%, $H^2$ = . 0.11 [ 0.04, 0.18]
Test of $\theta_i = \theta_j$: Q(0) = -0.00, p = .
Pulex
Iran 0.00 [ -0.00, 0.01] 7.44
Morocco 0.00 [ -0.01, 0.01] 6.96
Tunisia 0.24 [ 0.09, 0.38] 0.50
Heterogeneity: $\tau^2$ = 0.00, $I^2$ = 80.94%, $H^2$ = 5.25 0.00 [ -0.01, 0.02]
Test of $\theta_i = \theta_j$: Q(2) = 10.49, p = 0.01
Rhipicephalus
Palestin 0.04 [ 0.02, 0.06] 6.21
Egypt 0.00 [ -0.01, 0.01] 6.55
Heterogeneity: $\tau^2$ = 0.00, $I^2$ = 92.26%, $H^2$ = 12.93 0.02 [ -0.02, 0.06]
Test of $\theta_i = \theta_j$: Q(1) = 12.93, p = 0.00
Xenopsylla
Egypt 0.02 [ 0.01, 0.03] 7.04
Palestin 0.57 [ 0.20, 0.94] 0.08
Heterogeneity: $\tau^2$ = 0.13, $I^2$ = 88.39%, $H^2$ = 8.61 0.27 [ -0.27, 0.80]
Test of $\theta_i = \theta_j$: Q(1) = 8.61, p = 0.00
0
.5
1
Random-effects DerSimonian–Laird model

**S3 Figure. The prevalence of Bartonella spp. in ectoparasites of EMRO-WHO countries based on the different genus of ectoparasites, a systematic review and meta-analysis of the studies of the seven countries (1990-2022).**

**S1 Table. Characteristics of included studies by the target population (human, animal, and ectoparasites) in a systematic review and meta-analysis of Bartonellosis in World Health Organization Eastern Mediterranean Region (1990-2022).**

| Country | Population | Conducted study Years | Test type | Kind of sample | Sample size | Positive result | Quality score | Reference |
|---|---|---|---|---|---|---|---|---|
| **Animals** | | | | | | | | |
| Iran | Camels | 2019 | PCR | Blood | 106 | 18 | 6 | (1) |
| Iran | Cat | | PCR | Saliva | 100 | 0 | 4 | (2) |
| | | | PCR | Blood | 100 | 0 | | |
| | | | PCR | Nail | 100 | 0 | | |
| Iraq | Cat | 2013 | Culture | Blood | 207 | 9 | 5 | (3) |
| Iraq | Dog | 2008 | IFA | Blood | 97 | 46 | 4 | (4) |
| | Jackal | | IFA | Blood | 57 | 23 | | |
| | Red fox | | IFA | Blood | 39 | 5 | | |
| | Dog | | PCR | Blood | 97 | 36 | | |
| | Jackal | | PCR | Blood | 57 | 8 | | |
| | Red fox | | PCR | Blood | 39 | 0 | | |
| Egypt | Rodent | 2000-2012 | PCR | Blood | 1086 | 78 | 5 | (5) |
| Iran | Rodent | 2018-2019 | PCR | Fecal | 100 | 17 | 5 | (6) |
| Iran | Cat | 2005-2007 | PCR | Saliva | 140 | 12 | 5 | (7) |
| | | | PCR | Nail | 140 | 0 | | |
| | | | PCR | Blood | 140 | 5 | | |
| Pakistan | Rodent | 2004-2007 | PCR | Blood | 20 | 0 | 5 | (8) |
| Egypt | Rodent | 2004-2007 | PCR | Blood | 143 | 55 | 5 | (8) |
| Morocco | Dog | 2020 | PCR | Blood | 158 | 0 | 3 | (9) |
| | Cat | | PCR | Blood | 130 | 39 | | |
| Tunisia | Camels | 2015-2017 | PCR | Blood | 412 | 15 | 6 | (10) |
| Iran | Dog | 2019 | IFA | Blood | 66 | 49 | 6 | (11) |
| | | | PCR | Blood | 66 | 16 | | |
| Saudi Arabia | Rodent | 2011-2012 | PCR | Tissue | 138 | 94 | 4 | (12) |
| Iran | Dog | 2020 | PCR | Blood | 98 | 12 | 5 | (13) |
| Iran | Camels | 2018 | PCR | Blood | 100 | 0 | 6 | (14) |
| Egypt | Sheep | 2019-2021 | PCR | Blood | 38 | 3 | 5 | (15) |
| | Horse | | PCR | Blood | 8 | 1 | | |
| | Goat | | PCR | Blood | 28 | 1 | | |
| | Donkey | | PCR | Blood | 22 | 2 | | |
| | Cattle | | PCR | Blood | 112 | 6 | | |

| | | | | | | | | |
|---|---|---|---|---|---|---|---|---|
| | Buffalo | | PCR | Blood | 26 | 3 | | |
| Palestine | Dog | 2014 | PCR | Blood | 110 | 1 | 5 | (16) |
| | Sheep | | PCR | Blood | 7 | 0 | | |
| | Goat | | PCR | Blood | 3 | 0 | | |
| | Camel | | PCR | Blood | 19 | 0 | | |
| Iran | Cat | 2012 | PCR | Nail | 70 | 5 | 6 | (17) |
| | | | PCR | Saliva | 70 | 1 | | |
| Saudi Arabia | Cat or Dog | 1995-1997 | PCR | Blood | 188 | 5 | 5 | (18) |
| Tunisia | Dog | 1960-1995 | IFA | Blood | 149 | 51 | 6 | (19) |
| | | | PCR | Blood | 149 | 22 | | |
| Tunisia | Rodent | 1960-1995 | PCR | Blood | 383 | 188 | 5 | (20) |
| Egypt | Cat | 2016-2018 | PCR | Blood | 75 | 6 | 5 | (21) |
| Jordan | Cat | 2002 | IFA | Blood | 153 | 51 | 6 | (22) |
| Jordan | Cat | 2001-2003 | PCR | Blood | 20 | 4 | 6 | (23) |
| Egypt | Cat | 2008-2009 | IFA | Blood | 178 | 105 | 5 | (24) |
| Morocco | Dog | 2006 | IFA | Blood | 147 | 56 | | (25) |
| Iran | Cat | 2005 | Culture | Blood | 100 | 0 | 5 | (26) |
| | | | IFA | Blood | 100 | 23 | | |
| Iran | Camels | 2014 | PCR | Blood | 200 | 0 | 4 | (27) |
| Tunisia | Rodent | 2019-2020 | PCR | Tissue | 100 | 2 | 5 | (28) |
| **Human** | | | | | | | | |
| Morocco | Endocarditis | 2009-2010 | IFA | Blood | 19 | 1 | 3 | (29) |
| | | | PCR | Blood | 19 | 0 | | |
| Saudi Arabia | Endocarditis | 2015-2019 | IFA | Blood | 340 | 1 | 5 | (30) |
| Egypt | Endocarditis | 2005-2009 | PCR | Blood | 150 | 0 | 4 | (31) |
| | Endocarditis | | IFA | Blood | 150 | 5 | | |
| | Endocarditis | | Culture | Valve | 33 | 0 | | |
| | Endocarditis | | PCR | Valve | 33 | 1 | | |
| Tunisia | Endocarditis | 1997-2003 | IFA | Blood | 40 | 13 | 6 | (32) |
| | | | PCR | Blood | 40 | 12 | | |
| Egypt | Endocarditis | 2010 | PCR | Valve | 57 | 2 | 4 | (33) |
| | Endocarditis | | PCR | Blood | 156 | 0 | | |
| | Endocarditis | | IFA | Blood | 156 | 4 | | |
| Iran | Endocarditis Negative | 2022 | PCR | Blood | 100 | 0 | 6 | (34) |
| Bahrain | HIV Patient | 1996 | IFA | Blood | 56 | 9 | 5 | (35) |
| Egypt | Cat owners | 2016-2018 | IFA | Blood | 100 | 46 | 5 | (21) |
| Jordan | Children Randomly From the Hospital | 2001-2003 | IFA | Blood | 482 | 73 | 6 | (23) |
| Iran | Cat owners | 2005 | IFA | Blood | 100 | 18 | 5 | (26) |
| | **Ectoparasites** | | | | | | | |
| Morocco | Ctenocephalides spp and pulex of | 2007-2008 | PCR | Flea | 449 | 7 | 4 | (36) |

| | | | | | | | | |
|---|---|---|---|---|---|---|---|---|
| | Goat, Sheep and Cat | | | | | | | |
| Palestine | Ctenocephalides and Xenopsylla of rats, cat, and dog | 2011-2012 | PCR | Tick | 289 | 64 | 6 | (37) |
| Pakistan | Hyalomma of Cattle, Buffaloe | 2017 | PCR | Tick | 234 | 1 | 5 | (38) |
| Iran | Pulex of Human | 2019-2020 | PCR | Flea | 1937 | 10 | 5 | (39) |
| Tunisia | Pulex of Cat, Dog, Sheep, Goat | 2017 | PCR | Flea | 866 | 121 | 5 | (40) |
| Tunisia | Hyalomma of Camel | 2015-2017 | PCR | Tick | 300 | 0 | 5 | (10) |
| Jordan | Flea of Dog | 2016 | PCR | Flea | 108 | 0 | 5 | (41) |
| Egypt | Polyplax of R. norvegicus | 2002-2003 | PCR | Lice | 163 | 15 | 6 | (42) |
| Afghanistan | Fleas of Gerbil and Rattus spp. | 2002 | PCR | Flea | 77 | 20 | 6 | (43) |
| Egypt | Rhipicephalus of Cattle, Goat, Buffaloe, Donkey and Horse | 2019-2021 | PCR | Flea | 115 | 0 | 5 | (15) |
| Palestine | Rhipicephalus, Hyalomma and Haemaphysalis Of Dog, Sheep, Goat And camel | 2014 | PCR | Tick | 633 | 25 | 5 | (16) |
| Lebanon | C.felis of cat and Dog | 2011 | PCR | Flea | 155 | 3 | 4 | (44) |
| Egypt | Xenopsylla and Leptopsylla of R. rattus | 2002-2003 | PCR | Flea | 936 | 21 | 5 | (45) |

**Table S 2. Graphical report of quality assessment of included studies in the systematic review and meta-analysis studies of Bartonellosis in World Health Organization Eastern Mediterranean Region (1990-2022).**

| Country | The eligibility criteria | A clear definition of outcome | Description of locations, settings | Defination of Bartonella species | Sample size was arrived | Report of the number of outcomes | Quality score | Reference |
|---|---|---|---|---|---|---|---|---|
| Iran | ✅ | ✅ | ✅ | ✅ | ✅ | ✅ | 6 | (1) |
| Iran | ❌ | ✅ | ❌ | ✅ | ✅ | ❌ | 4 | (2) |
| Iraq | ✅ | ❌ | ✅ | ✅ | ✅ | ✅ | 5 | (3) |
| Iraq | ✅ | ❌ | ✅ | ✅ | ✅ | ❌ | 4 | (4) |
| Egypt | ✅ | ✅ | ✅ | ✅ | ❌ | ✅ | 5 | (5) |
| Iran | ✅ | ✅ | ✅ | ✅ | ❌ | ✅ | 5 | (6) |
| Iran | ❌ | ✅ | ✅ | ✅ | ✅ | ✅ | 5 | (7) |
| Pakistan | ✅ | ✅ | ✅ | ❌ | ✅ | ✅ | 5 | (8) |
| Egypt | ✅ | ✅ | ✅ | ❌ | ✅ | ✅ | 5 | (8) |

| | | | | | | | | |
|---|---|---|---|---|---|---|---|---|
| Morocco | ❌ | ❌ | ✅ | ✅ | ✅ | ❌ | 3 | (9) |
| Tunisia | ✅ | ✅ | ✅ | ✅ | ✅ | ✅ | 6 | (10) |
| Iran | ✅ | ✅ | ✅ | ✅ | ✅ | ✅ | 6 | (11) |
| Saudi Arabia | ✅ | ✅ | ✅ | ❌ | ✅ | ❌ | 4 | (12) |
| Iran | ✅ | ❌ | ✅ | ✅ | ✅ | ✅ | 5 | (13) |
| Iran | ✅ | ✅ | ✅ | ✅ | ✅ | ✅ | 6 | (14) |
| Egypt | ✅ | ✅ | ✅ | ❌ | ✅ | ✅ | 5 | (15) |
| Palestine | ✅ | ✅ | ✅ | ❌ | ✅ | ✅ | 5 | (16) |
| Iran | ✅ | ✅ | ✅ | ✅ | ✅ | ✅ | 6 | (17) |
| Saudi Arabia | ✅ | ❌ | ✅ | ✅ | ✅ | ✅ | 5 | (18) |
| Tunisia | ✅ | ✅ | ✅ | ✅ | ✅ | ✅ | 6 | (19) |
| Tunisia | ✅ | ✅ | ✅ | ❌ | ✅ | ✅ | 5 | (20) |
| Egypt | ✅ | ❌ | ✅ | ✅ | ✅ | ✅ | 5 | (21) |
| jordan | ✅ | ✅ | ✅ | ✅ | ✅ | ✅ | 6 | (22) |
| jordan | ✅ | ✅ | ✅ | ✅ | ✅ | ✅ | 6 | (23) |
| Egypt | ✅ | ✅ | ✅ | ❌ | ✅ | ✅ | 5 | (24) |
| Morocco | ✅ | ❌ | ✅ | ✅ | ✅ | ❌ | 4 | (25) |
| Iran | ✅ | ✅ | ❌ | ✅ | ✅ | ✅ | 5 | (26) |
| Iran | ✅ | ❌ | ✅ | ❌ | ✅ | ✅ | 4 | (27) |
| Tunisia | ✅ | ✅ | ✅ | ❌ | ✅ | ✅ | 5 | (28) |
| Morocco | ✅ | ❌ | ✅ | ❌ | ✅ | ❌ | 3 | (29) |
| Saudi Arabia | ✅ | ❌ | ✅ | ✅ | ✅ | ✅ | 5 | (30) |
| Egypt | ❌ | ✅ | ✅ | ❌ | ✅ | ✅ | 4 | (31) |
| Tunisia | ✅ | ✅ | ✅ | ✅ | ✅ | ✅ | 6 | (32) |
| Egypt | ✅ | ❌ | ✅ | ✅ | ✅ | ❌ | 4 | (33) |
| Iran | ✅ | ✅ | ✅ | ✅ | ✅ | ✅ | 6 | (34) |
| Bahrain | ❌ | ✅ | ✅ | ✅ | ✅ | ✅ | 5 | (35) |
| Morocco | ✅ | ❌ | ✅ | ✅ | ✅ | ❌ | 4 | (36) |
| Palestine | ✅ | ✅ | ✅ | ✅ | ✅ | ✅ | 6 | (37) |
| Pakistan | ✅ | ✅ | ✅ | ❌ | ✅ | ✅ | 5 | (38) |
| Iran | ✅ | ✅ | ✅ | ❌ | ✅ | ✅ | 5 | (39) |
| Tunisia | ✅ | ✅ | ✅ | ✅ | ✅ | ❌ | 5 | (40) |
| Jordan | ✅ | ✅ | ✅ | ❌ | ✅ | ✅ | 5 | (41) |
| Egypt | ✅ | ✅ | ✅ | ✅ | ✅ | ✅ | 6 | (42) |
| Afghanistan | ✅ | ✅ | ✅ | ✅ | ✅ | ✅ | 6 | (43) |
| Lebanon | ✅ | ❌ | ✅ | ✅ | ✅ | ❌ | 4 | (44) |
| Egypt | ✅ | ✅ | ✅ | ❌ | ✅ | ✅ | 5 | (45) |

1. Ghaemi M, Sharifiyazdi H, Heidari F, Nazifi S, Ghane MJVm. 'Candidatus Bartonella dromedarii'in the dromedary camels of Iran: Molecular investigation, phylogenetic analysis, hematological findings, and acute-phase proteins quantitation. 2019;237:108404.
2. Oskouizadeh K MB, Seyfiabad Shapouri M.R SK. A cross sectional study on Bartonella henselae infection in dogs in Ahvaz district by PCR. iranian Vaterinary journal. 2013;9(3):5-12.
3. Switzer AD, McMillan-Cole AC, Kasten RW, Stuckey MJ, Kass PH, Chomel BB. Bartonella and Toxoplasma infections in stray cats from Iraq. The American Journal of Tropical Medicine Hygiene. 2013;89(6):1219.
4. Chomel BB, McMillan-Cole AC, Kasten RW, Stuckey MJ, Sato S, Maruyama S, et al. Candidatus Bartonella merieuxii, a potential new zoonotic Bartonella species in canids from Iraq. PLoS Neglected Tropical Diseases. 2012.
5. Alsarraf M, Mohallal EM, Mierzejewska EJ, Behnke-Borowczyk J, Welc-Falęciak R, Bednarska M, et al. Description of Candidatus Bartonella fadhilae n. sp. and Candidatus Bartonella sanaae n. sp.(Bartonellaceae) from Dipodillus dasyurus and Sekeetamys calurus (Gerbillinae) from the Sinai Massif (Egypt). Vector-Borne Zoonotic Diseases. 2017;17(7):483-94.
6. Azimi T, Azimi L, Fallah F, Pourmand MR, Dogaheh HP, Tabatabaei SR. Detection and distribution of zoonotic pathogens in wild Norway rats (Rattus norvegicus) from Tehran, Iran. New Microbes New Infections. 2021;42:100908.
7. Oskouizadeh K, Zahraei-Salehi T, Sj A. Detection of Bartonella henselae in domestic cats' saliva. Iran J Microbiol. 2010.
8. Inoue K, Maruyama S, Kabeya H, Hagiya K, Izumi Y, Une Y, et al. Exotic small mammals as potential reservoirs of zoonotic Bartonella spp. Emerging infectious diseases. 2009;15(4):526-32.
9. Chekli Z, Haddad N, Mellouki F, Rhallabi N, Boulouis H. First molecular detection of Bartonella spp. in stray cats and dogs in Morocco. International Journal of Infectious Diseases. 2020;101:543.
10. Selmi R, Said MB, Yahia HB, Abdelaali H, Boulouis H-J, Messadi L. First report on Bartonella henselae in dromedary camels (Camelus dromedarius). Infection, Genetics Evolution. 2020;85:104496.
11. Greco G, Sazmand A, Goudarztalejerdi A, Zolhavarieh SM, Decaro N, Lapsley WD, et al. High prevalence of Bartonella sp. in dogs from Hamadan, Iran. 2019;101(4):749.
12. Kleynhans DJ, Sarli J, Hatyoka LM, Alagaili AN, Bennett NC, Mohammed OB, et al. Molecular assessment of Bartonella in Gerbillus nanus from Saudi Arabia reveals high levels of prevalence, diversity and co-infection. Infection, Genetics Evolution. 2018;65:244-50.
13. Samsami S, Ghaemi M, Sharifiyazdi H. Molecular detection and phylogenetic analysis of 'Candidatus Bartonella merieuxii'in dogs and its effect on hematologic parameters. Comparative immunology, microbiology infectious diseases. 2020;72:101504.
14. Bahari A, Azami S, Goudarztalejerdi A, Karimi S, Esmaeili S, Chomel BB, et al. Focus: Zoonotic Disease: Molecular Detection of Zoonotic Pathogens in the Blood and Tissues of Camels (Camelus dromedarius) in Central Desert of Iran. The Yale Journal of Biology Medicine. 2021;94(2):249.
15. Abdullah HH, Elbayoumy MK, Allam AM, Ashry HM, Abdel-Shafy S. Molecular epidemiology of certain vector-borne bacterial microorganisms in domestic animals and their ectoparasites in Egypt. Tropical Animal Health Production. 2021;53:1-11.
16. Ereqat S, Nasereddin A, Vayssier-Taussat M, Abdelkader A, Al-Jawabreh A, Zaid T, et al. Molecular evidence of Bartonella species in ixodid ticks and domestic animals in Palestine. Frontiers in Microbiology. 2016;7:1217.
17. Fard RMN, Vahedi SM, Ashrafi I, Alipour F, Sharafi G, Akbarein H, et al., editors. Molecular identification and phylogenic analysis of Bartonella henselae isolated from Iranian cats based on gltA gene. Veterinary Research Forum; 2016: Faculty of Veterinary Medicine, Urmia University, Urmia, Iran.

18. Alanazi AD, Alouffi AS, Alyousif MS, Alshahrani MY, Abdullah HH, Abdel-Shafy S, et al. Molecular survey of vector-borne pathogens of dogs and cats in two regions of Saudi Arabia. Pathogens (Basel, Switzerland). 2020;10(1):25.
19. Belkhiria J, Chomel BB, Ben Hamida T, Kasten RW, Stuckey MJ, Fleischman DA, et al. Prevalence and potential risk factors for Bartonella infection in Tunisian stray dogs. Vector-Borne and Zoonotic Diseases. 2017;17(6):388-97.
20. Fichet-Calvet E, Jomâa I, Ben Ismail R, Ashford RW. Patterns of infection of haemoparasites in the fat sand rat, Psammomys obesus, in Tunisia, and effect on the host. Annals of tropical medicine and parasitology. 2000;94(1):55-68.
21. Sayed AS, Alsaadawy RM, Ali MM, El-Hamid A, Rawhia F, Baty RS, et al. Serological and Molecular Detection of Bartonella henselae in Cats and Humans from Egypt: Current Status and Zoonotic Implications. 2022;9:859104.
22. Al-Majali AM. Seroprevalence of and risk factors for Bartonella henselae and Bartonella quintana infections among pet cats in Jordan. Preventive veterinary medicine. 2004;64(1):63-71.
23. Al-Majali AM, Al-Qudah KM. Seroprevalence of Bartonella henselae and Bartonella quintana infections in children from Central and Northern Jordan. Saudi Med J. 2004;25(11):1664-9.
24. Al-Kappany Y, Lappin M, Kwok O, Abu-Elwafa S, Hilali M, Dubey J. Seroprevalence of Toxoplasma gondii and concurrent Bartonella spp., feline immunodeficiency virus, feline leukemia virus, and Dirofilaria immitis infections in Egyptian cats. The Journal of parasitology. 2011;97(2):256-8.
25. Henn JB, Vanhorn BA, Kasten RW, Kachani M, Chomel BBJTAjotm, hygiene. Antibodies to Bartonella vinsonii subsp. Berkhoffii in Moroccan dogs. 2006;74(2):222-3.
26. Oskoeizadeh K, Zahraei Salehi T, Aldavood SJ, Majlesi B, Ghaffari H, Ashrafi Tamami I, et al. Study in prevalence of Bartonella henselae infection in domestic cats from Tehran. Journal of Veterinary Research. 2008;63(3):183-9.
27. Sazmand A, Harl J, Eigner B, Hodžić A, Beck R, Hekmatimoghaddam S, et al. Vector-borne bacteria in blood of camels in Iran: new data and literature review. Comparative Immunology, Microbiology Infectious Diseases. 2019;65:48-53.
28. Selmi R, Belkahia H, Dhibi M, Abdelaali H, Lahmar S, Said MB, et al. Zoonotic vector-borne bacteria in wild rodents and associated ectoparasites from Tunisia. Infection, Genetics Evolution. 2021;95:105039.
29. Boudebouch N, Sarih Mh, Chakib A, Fadili S, Boumzebra D, Zouizra Z, et al. Blood culture–negative endocarditis, Morocco. Emerging infectious diseases. 2017;23(11):1908.
30. Barry M, Bari SA, Akhtar MY, Al Nahdi F, Erlandez R, Al Khushail A, et al. Clinical and microbiological characteristics of infective endocarditis at a cardiac center in Saudi Arabia. Journal of Epidemiology Global Health. 2021;11:435-43.
31. Enany M, Rizk H, El-Kholy A, Rahman EA, Heshmat H, Sorour K, et al. First Report of Zoonotic Endocarditis in Egypt: High Prevalence of Brucella in Culture-Negative Endocarditis. International Journal of Infectious Diseases. 2012;16:e394.
32. Znazen A, Rolain J-M, Hammami N, Kammoun S, Hammami A, Raoult D. High prevalence of Bartonella quintana endocarditis in Sfax, Tunisia. The American journal of tropical medicine hygiene. 2005;72(5):503-7.
33. El-Kholy AA, El-Rachidi NGE-d, El-Enany MG, AbdulRahman EM, Mohamed RM, Rizk HH. Impact of serology and molecular methods on improving the microbiologic diagnosis of infective endocarditis in Egypt. Infection. 2015;43:523-9.
34. Dirbazian A, Sadeghimanesh M, Morovvati A, Soleimani M, Mirjani R, Mousavi SHJIJoMM. Molecular Detection of Infectious Endocarditis (Bartonella quintana) Bacteria from Selected Military Hospitals. 2022;16(5):457-64.

35. Yousif A, Farid I, Baig B, Creek J, Olson P, Wallace M. Prevalence of Bartonella henselae antibodies among human immunodeficiency virus-infected patients from Bahrain. Clinical infectious diseases. 1996;23(2):398-9.
36. Boudebouch N, Sarih M, Beaucournu J, Amarouch H, Hassar M, Raoult D, et al. Bartonella clarridgeiae, B. henselae and Rickettsia felis in fleas from Morocco. Annals of Tropical Medicine Parasitology. 2011;105(7):493-8.
37. Nasereddin A, Risheq A, Harrus S, Azmi K, Ereqat S, Baneth G, et al. Bartonella species in fleas from Palestinian territories: prevalence and genetic diversity. Journal of Vector Ecology. 2014;39(2):261-70.
38. Ghafar A, Cabezas-Cruz A, Galon C, Obregon D, Gasser RB, Moutailler S, et al. Bovine ticks harbour a diverse array of microorganisms in Pakistan. Parasites vectors. 2020;13:1-15.
39. Seidi s, Tavassoli M, Malekifard f. Cross-sectional study of Bartonella, Rickettsia and Wolbachia by molecular method in Fleas Ctenocephalides canis and Pulex irritans from the West and Northwest of Iran. Journal of Ardabil University of Medical Sciences. 2020;20(4):505-18.
40. Zouari S, Khrouf F, M'ghirbi Y, Bouattour AJP. First molecular detection and characterization of zoonotic Bartonella species in fleas infesting domestic animals in Tunisia. Parasites Vectors. 2017;10(1):1-9.
41. Zurita A, Gutiérrez SG, Cutillas C. Infection Rates of Wolbachia sp. and Bartonella sp. in Different Populations of Fleas. Current microbiology. 2016;73(5):704-13.
42. Reeves WK, Szumlas DE, Moriarity JR, Loftis AD, Abbassy MM, Helmy IM, et al. LOUSE-BORNE BACTERIAL PATHOGENS IN LICE (PHTHIRAPTERA) OF RODENTS AND CATT LE FROM EGYPT. 2006;92(2):313-8.
43. Marié JL, Fournier PE, Rolain JM, Briolant S, Davoust B, Raoult D. Molecular detection of Bartonella quintana, B. Elizabethae, B. Koehlerae, B. Doshiae, B. Taylorii, and Rickettsia felis in rodent fleas collected in Kabul, Afghanistan. The American journal of tropical medicine and hygiene. 2006;74(3):436-9.
44. Mba PA, Marié J-L, Rolain J-M, Davoust B, Beaucournu J-C, Raoult D, et al. Rickettsia felis and Bartonella henselae in fleas from Lebanon. Vector-Borne Zoonotic Diseases. 2011;11(7):991-2.
45. Loftis AD, Reeves WK, Szumlas DE, Abbassy MM, Helmy IM, Moriarity JR, et al. Surveillance of Egyptian fleas for agents of public health significance: Anaplasma, Bartonella, Coxiella, Ehrlichia, Rickettsia, and Yersinia pestis. The American journal of tropical medicine hygiene. 2006;75(1):41-8.
